# Design and development of an open-source high-temperature FFF 3D printer for high-performance polymers

**DOI:** 10.1016/j.ohx.2026.e00807

**Published:** 2026-07-02

**Authors:** Charlotte Thompson, Luke Salisbury, Evan Garrison, Lillian DeJean, Santanu Kundu, Matthew W. Priddy

**Affiliations:** a Michael W. Hall School of Mechanical Engineering, Mississippi State University, Mississippi State, MS, 39762, USA; b Center for Advanced Vehicular Systems, Mississippi State University, Starkville, MS, 39759, USA; c Department of Aerospace Engineering, Mississippi State University, Mississippi State, MS, 39762, USA; d East Mississippi Community College, Mayhew, MS, 39759, USA; e Dave C. Swalm School of Chemical Engineering, Mississippi State University, Mississippi State, MS, 39762, USA

**Keywords:** 3D printer, Additive manufacturing, Open-source hardware, RepRap, ULTEM, PEEK, Thermoplastics

## Abstract

Various commercial systems have been developed for printing high-performance polymers such as polyetherimide (PEI) and polyetheretherketone (PEEK) for use in medical, aerospace, automotive, and electrical components.High-performance polymers offer improved chemical resistance, thermal resistance, and mechanical properties compared to commonly used FFF materials such as PLA, PETG, and ABS. However, high-performance polymers are difficult to print due to the high printing temperatures required, such as nozzle temperatures up to 450°C, chamber temperature of 150°C, and bed temperatures up to 170°C. Most commercial systems capable of fabricating these materials are cost-prohibitive and lack modularity for user customization or modifications. An open-source, high-temperature FFF 3D printer was designed and constructed for high-performance polymers to be easily constructed, highly modular, and user-friendly. The printer was built using readily available components and controlled with a Duet3D motherboard running RepRap firmware to be highly customizable. The frame was built with aluminum T-slot channels to allow for integration with sensors, such as additional thermocouples or cameras for robust process monitoring. The printer can accommodate a 500°C nozzle, 200°C bed, and 120°C chamber. The system was validated through successful fabrication of ULTEM 1010, ULTEM 9085, and PEEK components. Performance was evaluated using three ULTEM 1010 calibration cubes printed under optimized process parameters. Dimensional measurements were used to assess accuracy, precision, and repeatability of the printer.

## Graphical abstract

**Figure:**
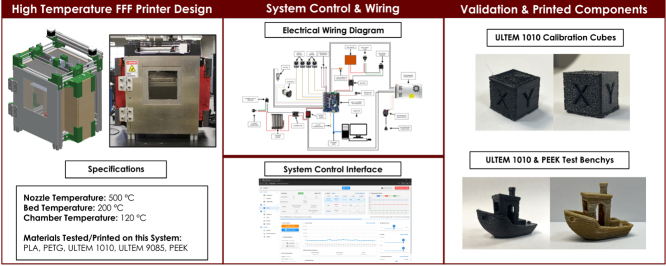

**Table:** 

**Specifications table**	

**Hardware name**	High Temperature FFF 3D Printer

**Subject area**	Educational tools and open source alternatives to existing infrastructure
**Hardware type**	Additive manufacturing
**Open source license**	CERN Open Hardware License Version 2 - Permissive
**Cost of hardware**	$2,664.80
**Source file repository**	https://doi.org/10.17605/OSF.IO/S9NVU

	

## 1 Hardware in context

Fused filament fabrication (FFF) is an additive manufacturing process in which material is deposited and fused in a layer-by-layer fashion to create a 3D object. Hotends are used in conjunction with extruders and nozzles to lay down material. In these systems, hotends heat the material above the glass transition temperature (Tg) for extrusion, while heated beds are maintained near Tg to promote adhesion to the build plate [1], [2], [3]. Tg is the temperature at which a material transitions from a hard, brittle, and glassy state to a softer, more malleable state where it may flow or warp [4]. FFF printers commonly use polymers such as thermoplastic filaments as the deposition material. Common thermoplastics used in 3D printing include polylactic acid (PLA), polyethylene terephthalate glycol (PETG), and acrylonitrile styrene (ABS). Thermoplastics can have either a semicrystalline or amorphous molecular structure. Semicrystalline thermoplastics have ordered molecular structures and defined melting temperatures, whereas amorphous thermoplastics have disordered structures and soften gradually over a range of temperatures. The chemical structure impacts Tg and coefficient of thermal expansion (CTE), which affects print strategy and mechanical properties of printed parts [5], [6]. Semicrystalline thermoplastics crystallize during cooling, causing greater shrinkage and warpage compared to amorphous thermoplastics in FFF printing [3].

FFF 3D printing follows a set of general steps in operation. First, a computer aided design (CAD) model must be made for the desired print and converted to a standard triangular language (STL) file, which is a triangular faceted approximation of the CAD file [7]. The generated STL file must then be imported into slicing software to create a geometric code (g-code). G-code is a computer numerical control (CNC) programming language that is used to drive manufacturing machines such as 3D printers [8]. These files are exported by a slicing software and contain information such as motion instructions, nozzle temperature, layer height, and flow rate of the material during printing. This file is then submitted to and read by the printer, which follows the g-code commands and moves to complete a print. When the printer reads the g-code file, it sets the temperatures needed for heating elements and creates the pattern of layer-by-layer motion needed to complete the print.

This additive manufacturing technique provides an accessible, low-cost method to create custom parts in small quantities for individual applications [9], [10], [11], [12]. FFF 3D printing is commonly used for prototyping to create low-cost models of parts for functionality or fit testing before investing in another manufacturing process to obtain a part with the needed material properties. Parts made with commonly used FFF materials can also be used for non-load bearing applications, such as wire harnesses and some small load-bearing parts such as brackets [13].

Based on the material used, some printers may use only a heated nozzle and bed, whereas others may require a heated chamber. For common materials such as PLA, PETG, and ABS, nozzle temperatures of 190-220°C, 220-260°C, and 210-265°C, respectively, are required. Generally, FFF 3D printers are capable of nozzle temperatures of 300°C and bed temperatures up to 100°C, which can comfortably print any of these common thermoplastics. The use of this method for fabrication of higher temperature materials, namely high-performance thermoplastics, has been explored in recent years. Some high-performance thermoplastics of interest for FFF currently are polyetheretherketone (PEEK) and polyetherimide (PEI or ULTEM). PEEK is a semicrystalline thermoplastic and PEI is amorphous. Compared to more common thermoplastics, PEEK and PEI require more robust systems capable of nozzle temperatures up to 420°C [14]. In addition to high-temperature nozzles, these materials also require a heated bed that can reach up to 150°C, and a heated chamber [15] capable of reaching and maintaining up to 200°C [13]. These higher temperatures and elevated heating elements are required for PEEK and PEI due to the higher Tg of 143°C [16] and 217°C [13], melting points at 334-340°C [16] and 330-380°C [13] respectively, and low coefficients of thermal expansion (CTE) compared to common thermoplastics. Due to these elevated thermal properties, high-performance thermoplastics frequently encounter issues in FFF printing, such as poor interlayer adhesion and delamination due to larger thermal gradients and CTE mismatch [1], [13], [17]. Elevated chamber temperatures mitigate these issues by providing sufficient heat to the material during printing, such that the thermal gradient and CTE mismatch between the deposited part and the build plate are minimized [18].

The use of FFF to manufacture these high-performance thermoplastics is attractive for many applications within aerospace [15], biomedical [19], [20], and electrical industries. Through this additive manufacturing method, these industries can create components with material properties comparable to metals on demand, cost effectively, and with full design freedom [21]. PEEK is an especially attractive material in the biomedical field for the fabrication of implants due to its mechanical strength, thermal stability, chemical stability [22], and ability to be sterilized by autoclaving [16]. On the other hand, PEI is more commonly used for structural applications due to its higher Tg, which allows for high thermal resistance and mechanical strength. There is a lack of understanding correlating printing parameters in FFF 3D printing to mechanical properties for PEI materials, especially with low-cost systems. There are a few studies of mechanical testing and properties for PEI through varied FFF 3D printing parameters in published literature, though these studies were done with the use of expensive industrial 3D printers such as an AON M2 and Fortus 450mc [23], [24], [25]. However, more documentation is available using both industrial and custom-built printers for PEEK and can be used to guide testing of print conditions in relation to yielded mechanical properties [26], [27], [28], [29].

Commercial FFF 3D printers capable of printing PEI, PEEK, and similar high-performance materials can range from $2600–50,000. Very few options are available under $5000, and these lower cost systems are not modular. A common trend in these systems is that the price generally increases with increased temperature capabilities and build volume as shown in Table 1. In addition to the high cost, commercial systems do not allow for the degree of modularity needed to adjust printing parameters and test various conditions such as chamber and bed temperatures above 120°C and 200°C respectively.

There are three notable open-source high-temperature FFF 3D printers in published literature to date [30], [31], [32]. The first was developed by NASA in 2016; the paper detailed the modification of a Lulzbot Taz 4 3D printer for fabrication of ULTEM 1010 by adding an enclosure and moving sensitive electrical components outside the heated area [30]. They also used PEI/ULTEM parts to replace ABS components, replaced the 300°C hotend with one capable of 400°C, and installed 12 IR heating lamps to surround the print and maintain higher temperatures during printing. Forced air convection and cooling shields were used to keep the motors from overheating when exposed to the higher chamber temperatures. Next, in 2020, Skrzypczak et al. at Michigan Technological University developed a high-temperature, open-source FFF 3D printer for under $1000 to fabricate heat-sterilizable personal protective equipment during supply shortages during the COVID-19 pandemic [31]. Their design included a 500°C hotend, 200°C heated bed, and enclosed chamber with a 1000 W space heater. All sensitive electrical components were removed from the heated area, and AC mains voltage was used for the heated bed and space heater. In another study conducted by Birkelid et al. an open-source liquid-cooled FFF 3D printer was developed in 2022 based on the hardware of an existing system for less than $1700 [32]. Birkelid et al. recognized the need for a lower-cost open-source system that would allow the user to more easily manipulate printing parameters to their needs and allow for the addition of sensors to monitor the printing process. With their modifications, the printer was able to reach a nozzle temperature of 500°C, bed temperature of 200°C, and chamber temperature of 135°C with all electronics inside the heated enclosure. This design also includes heated filament storage and a drying chamber. This printer was shown to successfully print parts with Carbon Fiber-PEEK.

**Table 1:** Available FFF 3D printers for high-performance materials.

Availability	Printer	Build Volume (mm)	Max Extruder Temperature (°C)	Max Bed Temperature (°C)	Max Chamber Temperature (°C)	Compatible Materials	Price (USD)
Commercial	Creatbot F160 (PEEK Version)	160 × 160 × 200	420	150	70	PLA, ABS, Nylon, PEEK	$2600
Commercial	Intamsys FunMat HT	260 × 260 × 260	450	160	90	PLA, ABS, Nylon, PEEK, PEKK, PPSU	$5995
Commercial	Apium P220	205 × 155 × 150	540	220	160	PEEK, PEI, Carbon Fiber PEEK	$30,000 -$50,000
Commercial	AON-M2	450 × 450 × 640	500	120	200	PEEK, PEI, Nylon, ABS	$49,999
Commercial	3ntr Spectral 30	300 × 300 × 300	500	Not Listed	250	PEEK, PAEK, PEI, PPSU	$100,000+
Non-Commercial	NASA 2016	298 × 275 × 250	400	200	N/A	ULTEM1010	Not Available
Non-Commercial	Skrypezak et al. 2020	Not Available	500	200	Enclosed	PEKK	under $1,000
Non-Commercial	Birkelid et al. 2022	500 × 500 × 500	500	200	135	Carbon Fiber-PEEK	$1700
Non-Commercial	Thompson et al. 2026	152 × 152 × 210	500	200	120	PLA, PETG, ULTEM 1010, ULTEM 9085, PEEK	$2664.80

For this project, a custom design was selected to provide flexibility and modularity without the constraints associated with modifying commercial systems designed for lower temperature operation. The goal of this project is to design and construct an open source, high-temperature FFF 3D printer for high-performance thermoplastic materials such as PEEK and PEI that is easily constructed, highly modular, and user friendly. This printer is designed to have capabilities to test high- and low-temperature spectrums for ULTEM 1010 material and allow for complete user control of printing parameter inputs.

**Fig. 1:**
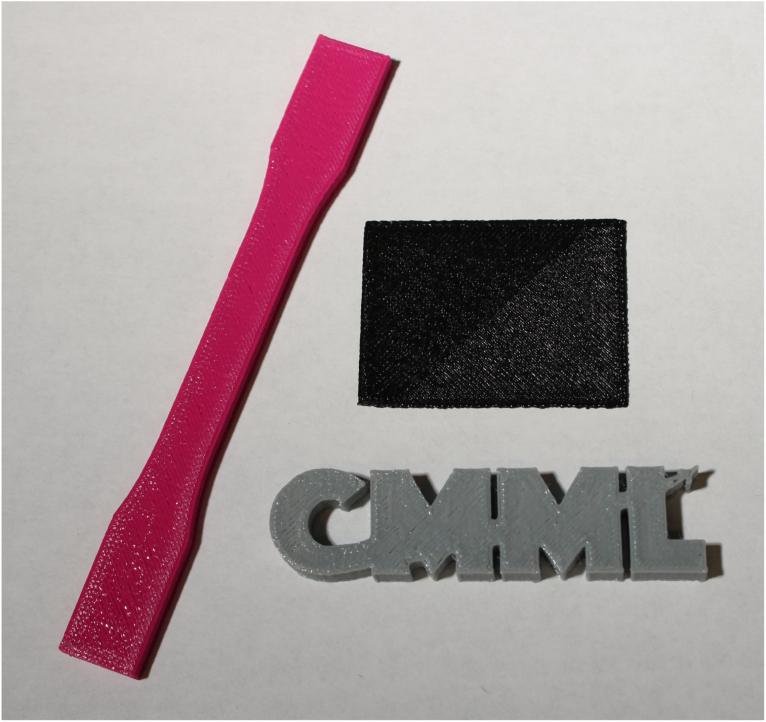
Test parts of PLA and PETG made with the high-temperature FFF printer during initial testing.

This project created a user-friendly, open-source option for high-temperature polymer 3D printing that allows for easy customization and operation in the fabrication of high-performance polymers. The printer was designed to be easily constructed by anyone who regularly uses a 3D printer and to test printing parameters of high-performance polymers. When testing with common thermoplastics (PLA+ and PETG), print parameters such as flow rate, layer height, print speed, line width, nozzle temperature, and Z-axis steps per mm were adjusted to obtain print quality and resolution comparable to a PRUSA MK3S+ as shown in Fig. 1. This high-temperature 3D printer was developed to print samples of ULTEM 1010 to draw correlations between print material, parameters, and material properties of the sample. The printer can be easily adapted for multiple applications, including:

• Prototyping and production of small-scale biomedical implants
• Prototyping and production of small-scale structural components for aerospace or automotive applications

## 2 Hardware description

**Fig. 2:**
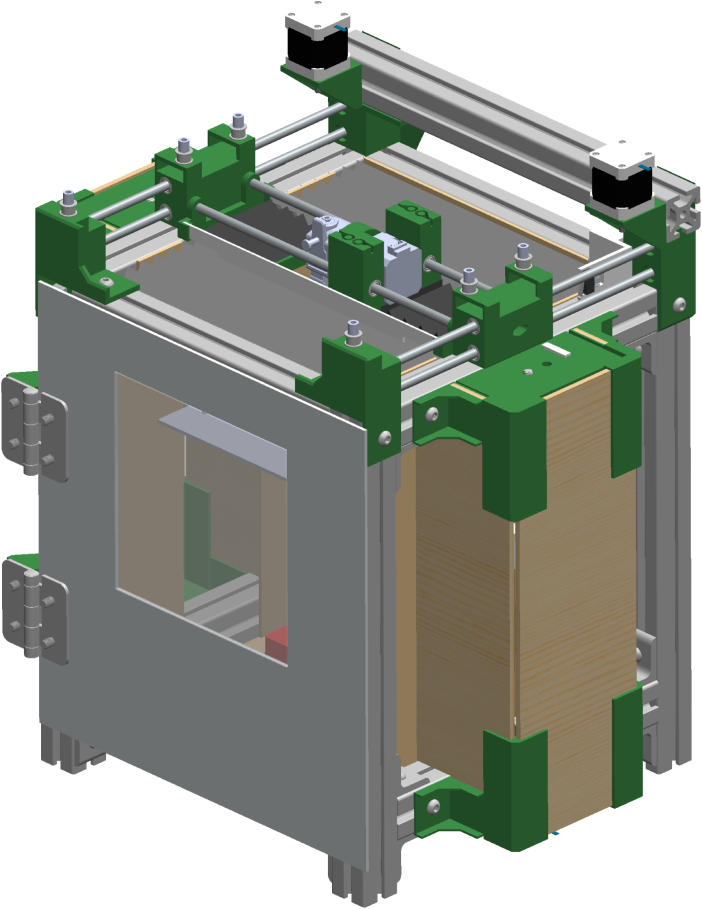
Isometric view of full printer assembly with 3D printed components in green.

Solid aluminum 40 mm T-slotted channels and corresponding corner brackets were used to construct the frame of the printer as shown in Fig. 2. The printer contains three main heating elements, a heated nozzle (500°C), heated bed (160°C), and chamber heater (165°C). The motion system of the printer nozzle was set up in a CoreXY configuration for improved speed and accuracy during printing. In this configuration the X- and Y-axis are controlled using two motors turning in tandem. This also allowed for ease of repair and manual adjustments to the motion system during high-temperature activity as it is isolated from the heated area. The bed/build plate was designed to move in the Z-direction (moving up to the nozzle) and supported by two aluminum t-slotted rails underneath the support plate. A borosilicate glass plate is used as a build plate due to its very low coefficient of thermal expansion. This printer supports a build volume of 152 × 152 × 210 mm (LxWxH). 3D printed parts were created and used on this printer to provide the mounting hardware for all components of the motion system and endstop mounting as shown in Fig. 2.

### 2.1 Frame

**Fig. 3:**
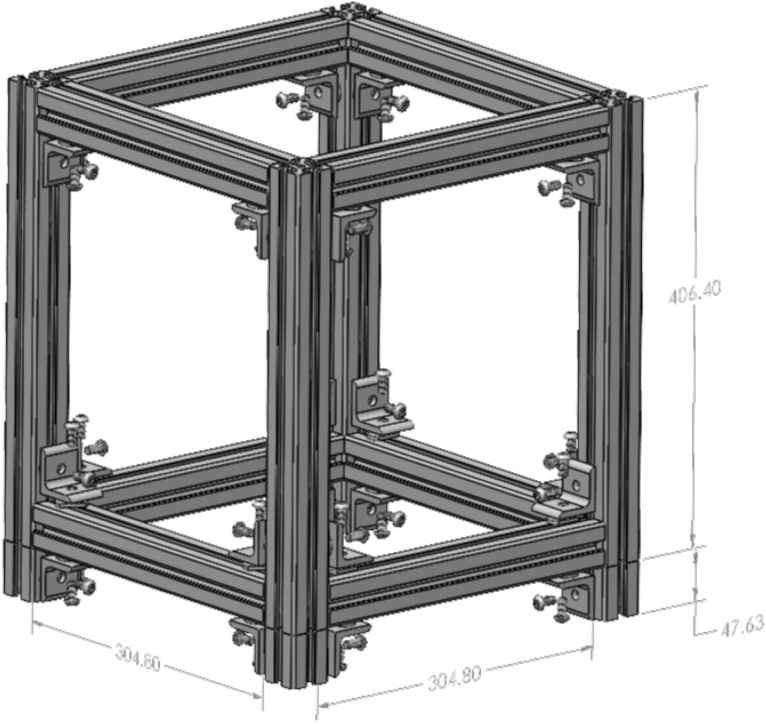
Isometric view of the printer frame made of 40 mm t-slotted rails, corner brackets, and fasteners with all dimensions in mm.

The frame of the printer was constructed with 16 pieces of 40 × 40 mm 6061 aluminum t-slotted framing rails as shown in Fig. 3. This size of framing rail was chosen for optimal rigidity in the printer during operation. The t-slotted rails were specifically chosen to facilitate the modularity needed to customize the motion system and enclosure design of the printer.

### 2.2 Heating elements

A Slice Engineering Mosquito Magnum + Air-Cooled Hotend allows for the printing of high-temperature thermoplastics reaching temperatures up to 490°C. This hotend was purchased pre-assembled and included two 50 W heaters as well as two PT1000 temperature sensors for temperature control and modulation.

A McMaster-Carr 6 × 6 in silicone heater allows for the control and modulation of bed heat temperatures up to 250°C. This heater requires 120VAC and 1.6 A and can generate 180 W of power. It has a watt density of 5 W/sqr in.

A PTC Ceramic Air Heater generates additional heat through convection inside the chamber to maintain heat within the build area. This heater has an optional voltage requirement of 110 V/220 V and can output 1000 W of power. For this work, 110VAC is used and that the heater output is 500 W. It has been paired with a large computer fan to distribute heat throughout the chamber.

### 2.3 Insulation and heat retention

This project required a heated chamber with the capability to:

• Retain consistent heated air around the build area
• Allow for ease of manual adjustments along all axes of motion

The sides and bottom of the chamber consist of 13 plywood boards with internal surfaces covered by mineral wool insulation cut to size and attached using a vulcanized silicone sealant. The mineral wool is 25.4 mm thick and can withstand temperatures up to 650°C. It also has a low heat flow rate of 240 Joules at 25°C. This insulation was selected instead of alternatives with similar properties because it is easy to handle, flame-resistant, and non-reflective.

### 2.4 Motherboard and firmware

A Duet2 Ethernet Motherboard is used to control all of the motors, heaters, fans, and temperature sensors. It is paired with a custom RepRap Firmware v3.3 created via the open-source RepRap Configuration Tool. Through the firmware, the terminals for each heater and corresponding temperature sensor are defined. It also coordinates the digital output commands sent from the motherboards to the heaters, motors, and fans. Through the firmware interface, the user can control the status (off/active/standby) of each heater or “tool” and monitor the temperatures read by the sensors as well as view other printing parameters. All motors are connected to axis-designated pin terminals, with one motor each for the X and Y axes and two motors for the Z axis.

### 2.5 Temperature measurement

Two RTD PT1000 temperature sensors are attached to the pre-assembled hotend. However, RepRap firmware allows only one temperature sensor to be assigned to each heating element for temperature control. Therefore, only one of the RTD PT1000 sensors is connected to the motherboard and used to regulate the hotend temperature. A surface J-type thermocouple is used to monitor the bed temperature and is mounted between the silicone heater and the aluminum build plate. A spot welded non-contact K-type thermocouple is routed through a gap at the bottom of the Z-axis enclosure on the right-hand side. The thermocouple is positioned so that it is approximately 3 inches (about 76 mm) above the heater in the middle of the stream to regulate the ambient temperature supplied by the chamber heater.

## 3 Design files

### 3.1 Design files summary

Table 2 lists the design files for all 3D printed parts used in the design and build of this printer. The majority of these parts consist of custom mounts and other parts for the CoreXY motion system. The RepRap firmware configuration files used to run this system are also included in this table.

**Table 2:** Design files overview.

Design filename	File type	Open source license	Location of file
Door Bracket	STL, STEP, SLDPRT	CERN-OHL-P V2	https://doi.org/10.17605/OSF.IO/S9NVU
CoreXY Linear Rod Support Back Left	STL, STEP, SLDPRT	CERN-OHL-P V2	https://doi.org/10.17605/OSF.IO/S9NVU
CoreXY Linear Rod Support Back Right	STL, STEP, SLDPRT	CERN-OHL-P V2	https://doi.org/10.17605/OSF.IO/S9NVU
CoreXY Linear Rod Support Front Left	STL, STEP, SLDPRT	CERN-OHL-P V2	https://doi.org/10.17605/OSF.IO/S9NVU
CoreXY Linear Rod Support Front Right	STL, STEP, SLDPRT	CERN-OHL-P V2	https://doi.org/10.17605/OSF.IO/S9NVU
CoreXY Carriage Frame Left	STL, STEP, SLDPRT	CERN-OHL-P V2	https://doi.org/10.17605/OSF.IO/S9NVU
CoreXY Carriage Frame Right	STL, STEP, SLDPRT	CERN-OHL-P V2	https://doi.org/10.17605/OSF.IO/S9NVU
CoreXY Idler Riser	STL, STEP, SLDPRT	CERN-OHL-P V2	https://doi.org/10.17605/OSF.IO/S9NVU
CoreXY Motor Mount	STL, STEP, SLDPRT	CERN-OHL-P V2	https://doi.org/10.17605/OSF.IO/S9NVU
CoreXY Axis Servo Mount	STL, STEP, SLDPRT	CERN-OHL-P V2	https://doi.org/10.17605/OSF.IO/S9NVU
CoreXY Extruder Hotend and Belt Holder Mount	STL, STEP, SLDPRT	CERN-OHL-P V2	https://doi.org/10.17605/OSF.IO/S9NVU
Z-axis Screw and Linear Rod Stabilizer Left	STL, STEP, SLDPRT	CERN-OHL-P V2	https://doi.org/10.17605/OSF.IO/S9NVU
Z-axis Screw and Linear Rod Stabilizer Right	STL, STEP, SLDPRT	CERN-OHL-P V2	https://doi.org/10.17605/OSF.IO/S9NVU
Z-axis Limit Switch Mount	STL, STEP, SLDPRT	CERN-OHL-P V2	https://doi.org/10.17605/OSF.IO/S9NVU
Z-axis Left Motor Mount	STL, STEP, SLDPRT	CERN-OHL-P V2	https://doi.org/10.17605/OSF.IO/S9NVU
Z-axis Right Motor Mount	STL, STEP, SLDPRT	CERN-OHL-P V2	https://doi.org/10.17605/OSF.IO/S9NVU
Enclosure Top Cover Attachment Adapter	STL, STEP, SLDPRT	CERN-OHL-P V2	https://doi.org/10.17605/OSF.IO/S9NVU
Enclosure Removable Top Cover Mount	STL, STEP, SLDPRT	CERN-OHL-P V2	https://doi.org/10.17605/OSF.IO/S9NVU
Enclosure Side Inter-Wall Brackets	STL, STEP, SLDPRT	CERN-OHL-P V2	https://doi.org/10.17605/OSF.IO/S9NVU
Enclosure Side Attachment Bracket	STL, STEP, SLDPRT	CERN-OHL-P V2	https://doi.org/10.17605/OSF.IO/S9NVU
Ambient Heater Interface Bottom-Side	STL, STEP, SLDPRT	CERN-OHL-P V2	https://doi.org/10.17605/OSF.IO/S9NVU
Ambient Heater Interface Top-side	STL, STEP, SLDPRT	CERN-OHL-P V2	https://doi.org/10.17605/OSF.IO/S9NVU
Ambient Heater Interface Dowel	STL, STEP, SLDPRT	CERN-OHL-P V2	https://doi.org/10.17605/OSF.IO/S9NVU
Full assembly	STL, STEP, SLDPRT	CERN-OHL-P V2	https://doi.org/10.17605/OSF.IO/S9NVU
System Control	RepRap File	MIT	https://doi.org/10.17605/OSF.IO/S9NVU

## 4 Bill of materials summary

[NOTE] Before construction, make note of the contents of the Appendix including warnings, repository contents, consumables, and needed tools for the printer developed in this paper. Table 3 summarizes the required parts and the cost of the system.

### 4.1 3Dprinted parts

The 3D printed components for this system were made with ABS model material by a Bambu Lab X1-Carbon 3D printer with a 0.4 mm nozzle. The print settings for each part were set to 40% infill density with an adaptive cubic infill pattern. For all 3D printed parts, the tree strong breakaway support style was selected.

**Table 3:** Bill of materials summary.

Component	Qty	Cost per unit (USD)	Total cost (USD)	Source of materials	Material type
Mosquito Magnum + Hotend	1	399.99	399.99	Slice Engineering	Non-specific
Chamber Heater	1	29.77	29.77	Amazon	Non-specific
Silicone Bed Heater	1	95.24	95.24	McMaster-Carr	Non-Specific
Hotend Fan	2	14.99	29.98	3D Labs	Non-specific
Bondtech Extruder	1	155.00	155.00	Slice Engineering	Non-specific
Vanadium Nozzle	1	21.99	21.99	Slice Engineering	Metal
Nano-Polymer Adhesive	1	45.00	45.00	Slice Engineering	Polymer
Duet 2 Ethernet Motherboard	1	205.70	205.70	Matterhackers	Non-specific
Duet v1.1 Thermocouple Daughterboard	1	39.99	39.99	Filastruder	Non-specific
Mechanical Limit Switches	1 pkg of 6	10.99	10.99	Amazon	Non-specific
Mineral Wool Insulation	1	12.83	12.83	McMaster-Carr	Inorganic
Wood Panels	2	7.15	14.30	Lowes	Organic
Silicone Adhesive	2	81.30	162.60	McMaster-Carr	Polymer
Bellows	2	30.92	61.84	McMaster-Carr	Polymer
Aluminum 6560 T-Slot Rails 40 × 40 mm (4ft)	4	48.00	192.00	McMaster-Carr	Metal
Aluminum 6063 T-Slot Rails 1” × 1” (4ft)	1	27.03	27.03	McMaster-Carr	Metal
Aluminum 7075 Block 1&1/2” × 4” × 6”	1	135.90	135.90	McMaster-Carr	Metal
40 mm T-Slot Brackets	3 pkgs of 10	18.99	56.97	Amazon	Metal
T-Slotted Framing Nut and M8 Button Head	27 pkgs of 4	2.72	73.44	McMaster-Carr	Metal
M5 flanged fasteners	3 pkgs of 4	4.63	13.89	McMaster-Carr	Metal
M5-.80 × 30 mm bolts	1 pkg of 7	2.48	2.48	Lowes	Metal
M5-.80 × 35 mm bolts	1 pkg of 7	2.48	2.48	Lowes	Metal
M5-.80 Nylon Nut	1 pkg of 6	1.98	1.98	Lowes	Metal
5 mm Washers	1 pkg of 20	2.28	2.28	Lowes	Metal
M3 × 40 mm bolts	1 pkg of 100	13.09	13.09	McMaster-Carr	Metal
M3-0.5 × 12 mm bolts	3 pkgs of 12	2.48	7.44	Lowes	Metal
M3-.50 nylon locking nuts	2 pkgs of 8	1.98	3.96	Lowes	Metal
M3 Washers	2 pkgs of 25	2.28	4.56	Lowes	Metal
M3 (bed-leveling) screws and springs	1 pkg of 4	8.99	8.99	Amazon	Metal
M4 -.70 × 12 mm bolts	1 pkg of 12	2.48	2.48	Lowes	Metal
Linear Rods	5 pkgs of 2	12.99	64.95	Amazon	Metal
Lead Screws and Nuts	1 pkg of 2	13.99	13.99	Amazon	Metal
Flexible Shaft Couplings 5 mm to 8 mm	1 pkg of 5	9.99	9.99	Amazon	Metal
Stepper Motors	4	16.27	65.08	StepperOnline	Non-specific
Belts & Toothed Idlers	1	14.99	14.99	Amazon	Polymer
Smooth Idlers	1 pkg of 20	11.99	11.99	Amazon	Metal
Linear Ball Bearings	1 pkg of 12	11.99	11.99	Amazon	Metal
Glass Bed Clamps	1 pkg of 8	9.99	9.99	Amazon	Metal
Aluminum Bed Spacers	4	2.07	8.28	McMaster-Carr	Metal
Borosilicate Glass Sheet 7” × 7” × 1/8” (build plate)	1	26.85	26.85	McMaster-Carr	Inorganic
Aluminum 6061 Sheet 1/8” Thick, 8” × 8” (heated bed and z-axis support)	2	15.97	31.94	McMaster-Carr	Metal
Aluminum 6061 Sheet 1/8” Thick, 18” × 18” (Door)	1	63.88	63.88	McMaster-Carr	Metal
Borosilicate Glass Sheet 8” × 8” × 1/8” (Window)	1	35.38	35.38	McMaster-Carr	Inorganic
PC Fan	1	13.99	13.99	Amazon	Non-specific
Solid State Relay	2	46.51	93.02	Amazon	Non-specific
DC Power Supply	1	33.50	33.50	Amazon	Non-specific
Power Supply Switch	1	7.99	7.99	Amazon	Non-specific
Power Supply Cord	1	11.98	11.98	Amazon	Non-specific
Power Cord (Chamber & Bed Heater)	2	13.99	27.98	Amazon	Non-Specific
MOSFET Board	1	15.56	15.56	Amazon	Non-specific
Button Circuit Breaker	2	6.99	13.98	Amazon	Non-specific
Gear Grease	1 pkg of 3	7.99	7.99	Amazon	Organic
Electrical Tape	1 pkg of 3	8.93	8.93	Amazon	Polymer
ABS Filament	3	19.99	59.97	BambuLab	Polymer
Hinge for 40 mm High Rail, 3” Long	2	22.84	45.68	McMaster-Carr	Metal
Loctite 4902	1	66.11	66.11	Amazon	Polymer
22 AWG Wire 25ft	1	9.98	9.98	Amazon	Metal
Ferrule Terminal Connectors 22 AWG	1 pkg of 1000	7.99	7.99	Amazon	Metal
Fork Terminal Connectors 22 AWG	1 pkg of 25	4.71	4.71	Digikey	Metal
Ferrule Terminal Connectors 14 AWG	1 pkg of 10	1.23	1.23	Digikey	Metal
Fork Terminals 14 AWG	1 pkg og 10	2.58	2.58	Digikey	Metal
14 AWG Wire 50ft	1	24.91	24.91	Grainger	Metal
Ring Terminal Connectors 14 AWG	1 pkg of 10	3.23	3.23	Digikey	Metal

**Total**			**$2664.80**		

### 4.2 Machined components

#### 4.2.1 Door

**Fig. 4:**
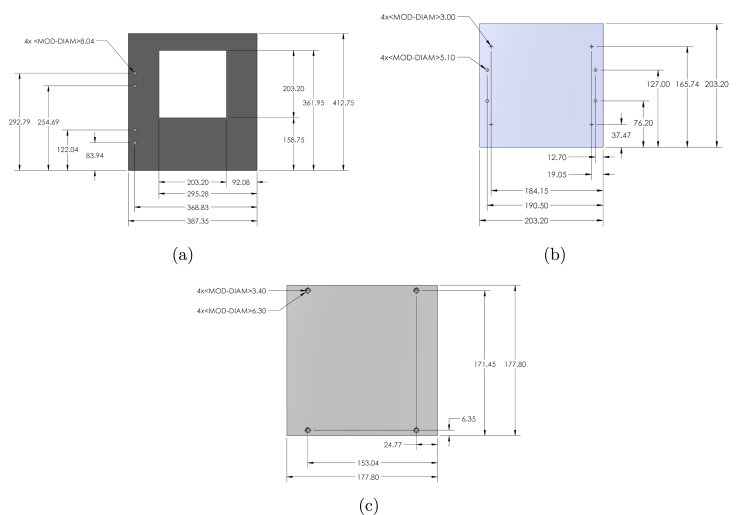
(a) Front view of the aluminum door with dimensions in mm. (b) Top view of aluminum support plate with hole diameters and outer dimensions in mm. (c) Top view of the aluminum heated bed plate with diameters and outer dimensions in mm.

The door is constructed from a 387.35 × 412.75 × 6.35 mm (16.25 × 15.25 × 0.25 in) 6061 aluminum plate with a window cut-out and through holes for mounting to the hinges. The window cut-out was completed with water jetting, and the through holes were cut with a drill press. The dimensions for these cuts are shown in Fig. 4a.

#### 4.2.2 Aluminum support plate

This 6061 aluminum plate is 203.2 × 203.2 × 1.375 mm (8 × 8 × 1/8 in) with four drilled and tapped holes machined closest to the corners of the plate. These four holes were drilled to 2.5 mm diameter with a mill and tapped for a M3 × 0.5 thread with a T-handle tap wrench. Four 5 mm through holes were also drilled to connect this plate to 25.4 mm (1 in) t-slot rails on the Z-axis as shown in Fig. 4b.

#### 4.2.3 Aluminum heated bed plate

This 6061 aluminum plate is 117.80 × 117.80 × 1.375 mm (7 × 7 × 1/8 in). Four 3 mm diameter through holes were machined into the plate with a mill. Then countersink holes were milled to 1.5 mm depth from the top of the plate with a 90°angle countersink bit such that 3–4 threads are left intact. Fig. 4c reflects the location and size of each hole machined on this plate.

#### 4.2.4 Aluminum T-slotted framing rails

17 pieces of 40 × 40 mm 6063 aluminum t-slotted rails were cut into various lengths to make up the frame of the printer using a bandsaw. The quantities and lengths of these pieces are as follows:

• 8x 304.8 mm (12 in) (top and bottom horizontal on sides and from and back)
• 4x 47.625 mm (small ones supporting feet on bottom and mounts on top)
• 4x 406.4 mm (tall vertical rails)
• 1x 385 mm (top back horizontal rail)

#### 4.2.5 Aluminum T-slots for Z-axis motion

Two 25.4 × 25.4 mm (1 × 1 in) 6061 aluminum t-slotted rails were used to support the Z-axis motion system of the printer. The two rails were cut to 417 mm in length.

#### 4.2.6 Aluminum Z-axis sliding mounts

**Fig. 5:**
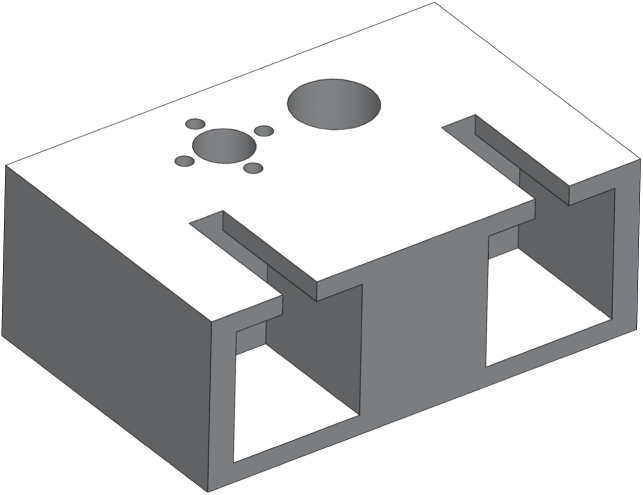
Isometric view of left Z-axis sliding mount.

These two 7075 aluminum mounts were machined using a CNC machine, each with 6 holes through the top to attach the lead screw and linear rods as well as two cutouts on the side face to provide a slot for the 25.4 × 25.4 mm (1 × 1 in) t-slotted rails to have a friction fit as shown in Fig. 5. A drawing with the dimensions of this part is available in the repository.

### 4.3 Custom components

#### 4.3.1 Wooden panels

The plywood is cut into 12 pieces with a table saw to form the chamber enclosure for the printer. Both the left and right sides of the enclosure consist of five panels, with the other two boards covering the back and bottom of the printer. The front panel for each side of the enclosure is 410 × 90 mm. For each board, two of the corners are cut out, such that the cutouts are on the same 410 mm sides and are 40 × 40 mm each. The enclosure has panels perpendicular to the frame in front of and behind the Z-axis rods on each side of the printer. Each of these four boards are 410 × 120 mm with the corners cut according to the prior specifications. The printer is enclosed on each side next to the Z-axis by 410 × 140 mm panels. The remainder of the left and right sides of the enclosure consists of two 410 × 130 mm boards with the corners removed in the same way as the other panels. A 410 × 310 mm panel covers the backside of the printer. This board has 40 × 40 mm squares cut from two corners on the same 310 mm side. The final panel is used to enclosed the bottom of the printer and is 310 × 310 mm with a 40 × 40 mm square cut from each corner. This panel also has a 100 × 100 mm hole cut approximately in the middle. For the foremost and backmost side panels, one hole in the top and bottom is drilled in each for attachment to the interior sides of the framing. Eight holes are drilled in the back and bottom panels, with two on each side. The back panel is attached to the framing rails similarly to the side panels on the inside face. However, the bottom panel is attached to the outer face of the t-slot framing from the chamber interior for ease of installation. All of the holes drilled are 9 mm for each of the panels to attach to the 40 mm t-slots with compatible fasteners.

#### 4.3.2 Mineral wool sheets

The mineral wool insulation sheet is cut into 15 pieces to cover the door/front, side, back, and bottom panels of the enclosure. A large flat surface that can be damaged, such as a piece of cardboard, and a box or insulation cutter are needed to cut this material. Mineral wool insulation can be easily cut into the desired shapes to cover the interior of the chamber. These pieces should be fashioned to cover all interior facing walls of the enclosure to ensure proper insulation. Proper personal protective equipment should be used when cutting this material. A well-ventilated room is required, though a mask, gloves, and safety glasses are also recommended.

## 5 Build instructions

**Fig. 6:**
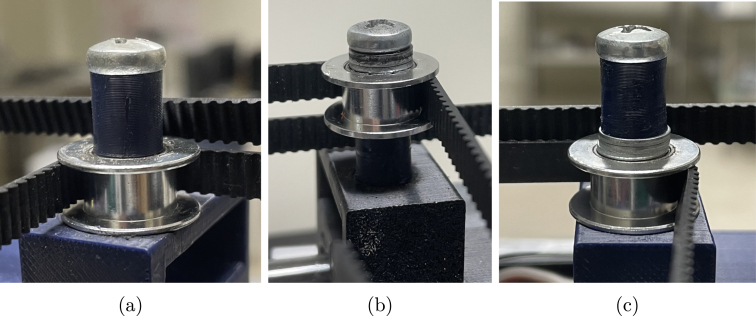
Smooth idler configurations on carriage frames.

**Fig. 7:**
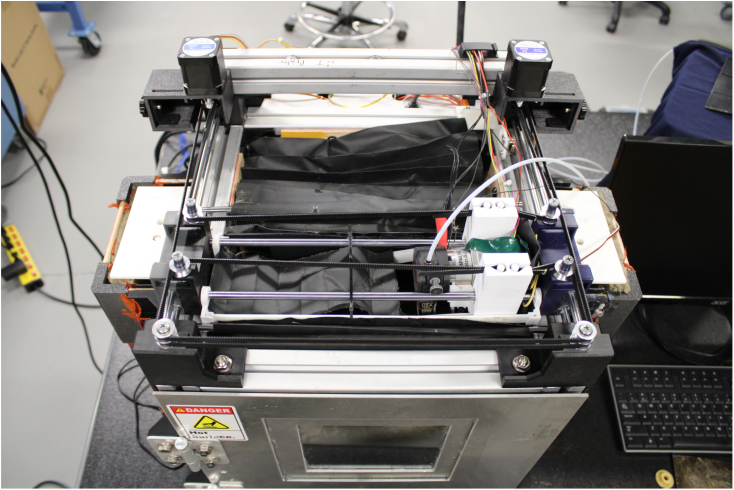
Top view showing the CoreXY motion system.

**Fig. 8:**
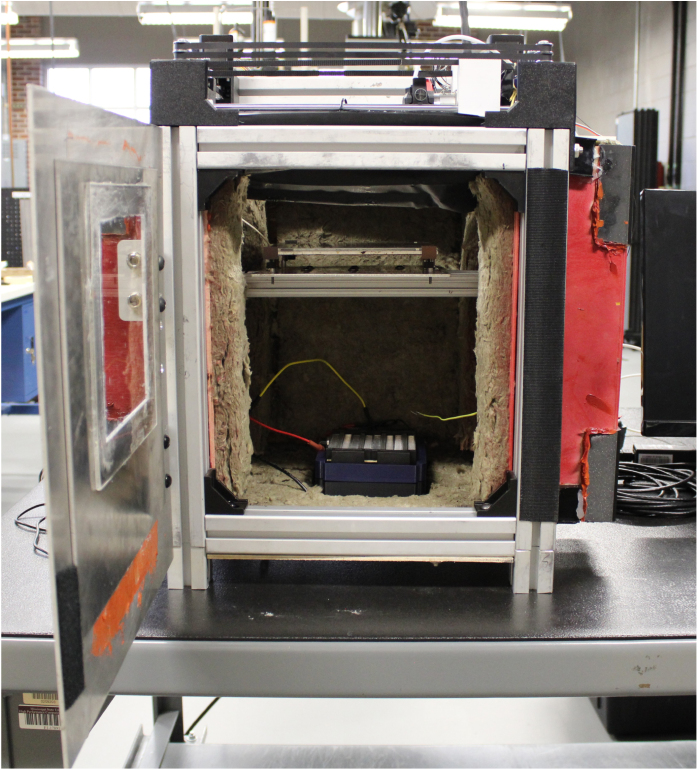
High temperature printer interior.

**Fig. 9:**
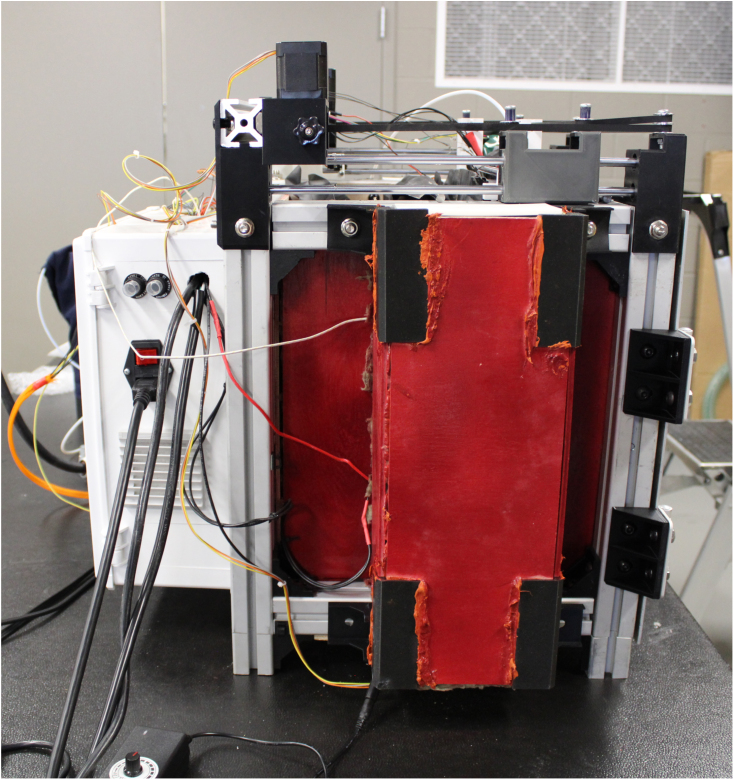
Side view of enclosure panels attached.

1. Assemble the frame in the configuration shown in Fig. 3 using the aluminum corner brackets and M8 fasteners. The nuts for these corner brackets should slide inside the slot of the framing rails and be placed behind the hole in each corner bracket so that they are aligned, allowing the screw to thread into the nut and securing the corner brackets in their designated locations. There are 28 corner brackets used to complete this frame configuration, and each uses two sets of M8 nuts and bolts. This totals to 56 sets of nuts and bolts. The brackets should be tightened into position and connected to the framing rails using a 5 mm Allen key and 13 mm wrench. During this process, decide on the front side of your printer. All directions following this step will be relative to the front face.
2. Attach a stepper motor to each of the motor mounts with four M3 stainless steel screws each. For the CoreXY motor mounts, two washers should be placed between the M3 screw and the motor mount. No washers are needed for the Z-axis motor fasteners.
3. Assemble the Z-axis motion system:
  (a) Secure both of the Z-axis motor mounts 95 mm (3 & 3/4 in) from the front face of the bottom t-slotted rails with two M8 nuts and bolts each.
  (b) Place the Z-axis limit switch mount into the right stabilizer and glue into place. Then attach the limit switch using M3 screws and M3-0.5 nylon nut with an adjustable wrench and phillips head screwdriver.
  (c) Place the Z-axis stabilizers 114.3 mm (4 & 1/2 in) from the inner side of the front face with one M8 screw and nut each. Attach them to the t-slotted frames loosely so they can slide. These will be fully tightened in a future step.
  (d) Place one shaft coupler on each of the motor shafts. Use a 2 mm Allen key to tighten one set screw along the flat side of the motor shaft. Make sure that the end of the motor shaft does not take up more than half of the coupler.
  (e) Place two 20 mm t-slotted rails into their friction fit slots on the inside of each Z-axis sliding mount. During this process, attach the T8 brass nut to each Z-axis sliding mount using M3 × 40 mm screws and M3-0.5 nylon locking nuts. Then, add one bearing into each through-hole beside the T8 brass nut. Using electrical tape as a gasket, the bearing should be friction-mounted into each Z-axis sliding mount.
  (f) Install the Z-axis support plate in the center of the 20 mm t-slots with four 5 mm (M5) flanged nuts and corresponding bolts. Tighten the 5 mm bolts to secure this support plate to the Z-axis t-slots with a 3 mm Allen key.
  (g) Place the Z-axis assembly so that the holes in both Z-axis sliding mounts align with the holes on the Z-axis linear stabilizer. After alignment, slide the threaded rod through the set of holes so that it rests in the shaft coupler. Use the 2 mm Allen key to tighten down the two set screws and secure the threaded rods on each side to the shaft coupler. Make sure that there is distance between the motor shaft and the threaded rod. Slide the smooth rod through the other hole, making sure it makes contact with the linear bearing and the motor mount.
4. Assemble the Build Plate:
  (a) Attach the heated bed with adhesive to the center of the bottom face of the Z-axis aluminum build support plate. The silicone heated bed should be positioned such that it is 1 in inside from all sides of the aluminum plate to supply heat to a proper 152 × 152 mm area on the top of the build plate.
  (b) Install the aluminum build support plate with the heated bed attached to the aluminum support plate with M3 × 35 mm adjustable leveling screws, springs, and spacers. The screws should be put through the through holes of the aluminum build support plate, then the springs and spacers should be put around the bottom of the screws just before they are threaded into the tapped holes in the aluminum support plate.
  (c) Attach the glass build plate on top of the aluminum build support plate with adjustable glass bed mounts on each corner. Metal binder clips may be used here temporarily for testing or manual bed leveling.
5. Assemble the CoreXY motion system:
  (a) Press fit one linear bearing into each slot of the extruder and hotend mount (for a total of two bearings used).
  (b) Begin by attaching two linear rods to the left carriage frame and sliding a singular top cover attachment adapter such that it is flush with the surface of the left carriage frame. Slide the extruder and hotend mount onto the frame. Finally, add the right top cover attaching adapter such that it faces the first one.
  (c) Attach the x-axis limit switch to the inside of the right carriage frame using two M3 × 15 mm screws and two M3-0.5. Cap the carriage linear rod stack by press fitting the finished right carriage into them.
  (d) Press fit two linear bearings into each hole of the left and right carriages (for a total of eight bearings used).
  (e) Attach the back right and back left linear rod support to each end of a 406 mm (16 in) long T-Slotted framing rail using M8 nuts and bolts. Attach 2 linear rods to each linear rod support.
  (f) Slide the carriage frame onto the y-axis linear rods and cap each set of rods with the front left and front right support.
  (g) Align the CoreXY motion system for the holes in the top of the structure frame and bolt the system to the frame using M8 nuts and bolts.
  (h) Move the x-axis configuration along the y-axis linear rods to ensure that they are even and the carriages glide freely.
  (i) Attach one toothed idler to each CoreXY motor shafts with a 1.5 mm Allen key.
  (j) Attach two smooth idlers (stacked) on the front left and right linear rod supports each (for four idlers used) using M5 × 30 mm screws and nuts. Then attach a smooth idler and spacer to the left carriage frame in its slot closest to the back face of the printer, and another in the foremost slot on the right carriage frame using M5 × 30 mm screws and nuts in the configuration shown in Fig. 6a. Finally, attach a smooth idler, three washers, and a spacer to the foremost slot on the left carriage frame and the slot closest to the back face on the right carriage frame as shown in Fig. 6b and Fig. 6c respectively. Be sure that the nylon nuts are tightened such that they are loose enough to allow for the idlers to turn smoothly.
  (k) Attach two belts of 1372 mm each (54 in) routed through the attached idlers in a traditional CoreXY configuration as shown in Fig. 7.
  (l) Attach the y-axis limit switch mount flush with the inside face of the back right CoreXY linear rod mount. Then attach the y-axis limit switch with two M3 × 12 mm screws and nylon nuts.
6. Assemble the Direct Drive (Hotend and Extruder):
  (a) Put one screw through one of the connecting holes with the hotend upside down so that the screw falls into place
  (b) Match up the filament extrusion exit hole with the top middle hole on the hotend while holding them upside down Using a 5/64” Allen key, turn the screw for a few threads to secure it very loosely
  (c) Put the second screw in place and screw it down a few threads as well
  (d) Continue tightening both screws until they are securely fastened. Once the hotend is secured to the extruder, the top face should be flush with the bottom face of the extruder and you should not be able to move the hotend or wobble it with your hand
7. Place the hotend and extruder down into its mount along the x-axis linear rails and tighten the top piece of the collar down to secure it in place. (Please note that the collar may be left off during manual bed leveling to ensure that the glass bed and hotend are not damaged in this process.)
8. Attach the 40 × 40 mm hotend fan to the right fan mount (built into the hotend and extruder mount) with four M3 × 12 mm screws and four M3 nuts. The second fan slot (on the left) may be left unused, as there is sufficient cooling with one fan. However, this was made to provide an option for additional cooling during printing.
9. Assemble the Enclosure:
  (a) Cut the mineral wool insulation into 13 sections (5 on each side, 1 back, 1 bottom, 1 door) and positioned on the enclosure panels as shown in Fig. 8. Follow the directions to cut the insulation in the custom components section.
  (b) Mineral wool insulation must be attached to the panels with silicone adhesive. The sealant is placed on the inside face of each panel in beads. The insulation sections must then be placed over these areas. Press down and hold the insulation in place for 5 min. Then flip the panel over and place some weight on the backside to allow the silicone adhesive to dry for 24 h.
  (c) Once the adhesive dries, the plywood boards are placed around the frame and Z-axis rods to cover the sides, back, front, and bottom of the printer. The panels surrounding the Z-axis are connected to each other by custom ABS brackets on the top and bottom. These brackets are held in place by the same silicone sealant. All of the boards are attached to the frame by 40 mm stainless steel t-slotted fittings, as shown in Fig. 9.
  (d) Use Loctite 4902 (or another super glue rated to 80°C or above) to glue the large cut, y-axis bellows to the removable top cover mount. After the glue has dried, place the removable top cover mount into the top cover attachment adapter on the front or back. Then glue the bellows to the side of the enclosure facing the attachment. Repeat for the other side. The side (x-axis) bellows are attached to the inside face of the top cover attachment adapter using glue, and clips or glue are used to connect them to the motor carriage.

### 5.1 Electronics assembly instructions

**Fig. 10:**
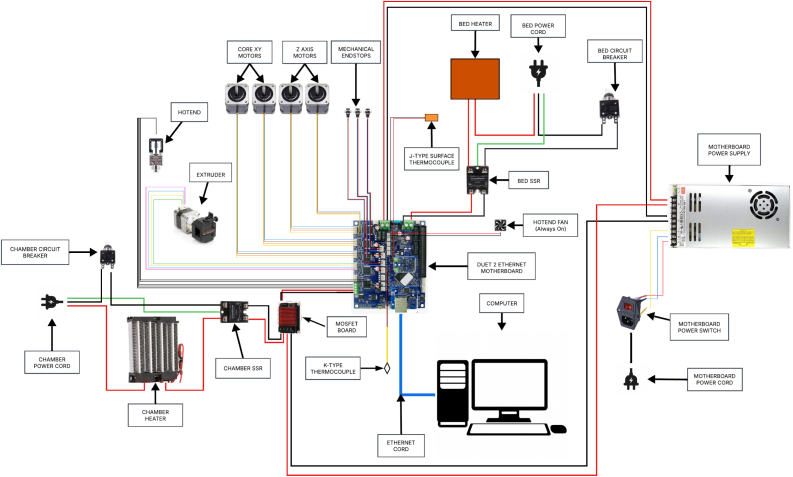
High temperature FFF printer wiring diagram.

Refer to Fig. 10 and the Duet 2 Wifi and Ethernet Hardware Documentation [33] throughout the electronics assembly.

[WARNING] The use of AC mains power can be dangerous if not handled or grounded properly.

(a) Attach the power supply to the “Power In” terminal on the Duet 2 Ethernet Motherboard and MOSFET board with 14 AWG gauge wire from open positive and negative terminals as shown in Fig. 10. Use the fork terminal connectors in the power supply terminals and 14 AWG ferrules for the motherboard and MOSFET terminals. Crimpers for these respective connector types should be used to secure the wire in the connector for best contact in with the terminals. All connections to or from the power supply use 14 gauge wire except connections to the power switch.
(b) Attach the MOSFET board to the E1 extruder heat terminal with 22 gauge wires and ferrules. Then run 22 gauge wire from “hotbed” terminals of the MOSFET to the input terminals of the chamber SSR. Use the 22 gauge fork terminal connectors on the SSR input.
(c) Run a 14 gauge wires from the output of the chamber SSR to the chamber circuit breaker and the chamber heater.
(d) Connect the chamber power cord live wire (white in-person, labeled red in Fig. 10) to the chamber heater. then connect the black wire to the chamber circuit breaker and connect the ground wire (green) to the chamber SSR using a 14 gauge ring terminal connector.
(e) Attach the bed heater SSR to the “Heated Bed” terminal on the motherboard using 22 gauge wire, ferrules, and fork connectors running from the input terminals of the SSR.
(f) Connect the bed heater SSR to the heated bed using a 14 gauge wire and fork terminal connector from the positive output terminal of the SSR. Then connect the negative terminal of the SSR output to the bed circuit breaker using more 14 gauge wire and another fork terminal on the SSR side.
(g) Attach the bed power cord live wire (white in-person, labeled red in Fig. 10) to the bed heater and the negative (black) to the bed circuit breaker. Then attach the ground wire (green) to the bed SSR using a 14 gauge ring terminal connector.
(h) Attach the thermocouple daughterboard to the designated expansion board pin terminals denoted by SPI0 on the motherboard.
(i) Attach limit switches in the X_STOP, Y_STOP, and Z_STOP terminals respectively. These connections use 3-way white Molex KK-type housings.
(j) Attach CoreXY and Z-axis motors to terminals X Stop, Y Stop, and Z Stop respectively. (Please note that there are two Z Stop terminals that are stacked on the motherboard to support two Z-axis motors.) The connections that come with these stepper motors are compatible with the motherboard pin terminals.
(k) Attach the hotend heater wires to E0 extruder heater terminal using 16–18 AWG gauge ferrule connectors. One wire from each heater on the hotend should go into the positive and negative terminals respectively such that two wires are in each ferrule connector for attachment. Polarity of the wires does not matter for these heaters.
(l) Attach the hotend thermistor wires to the E0 TEMP terminal using a 2-way white Molex KK-type housing included with the duet motherboard.
(m) Attach the extruder motor wires to the E0 stepper terminal using its given connector.
(n) Attach the 40 mm hotend fan to an “Always On” fan terminal using a 2-way white Molex KK-type housing.
(o) Attach the J-type surface thermocouple for the bed heater to the thermocouple daughterboard in terminal TC1. Then attach the K-type theromcouple for the chamber heater to the thermocouple daughterboard in termianl TC0.

### 5.2 Set up motherboard connection and firmware

(a) Building the Firmware and Printer Interface.
  i. The custom firmware configuration files for the printer detailed in this paper are supplied in the repository for this work. Using your chosen web browser, navigate to the RepRap config tool and choose the option to create a firmware configuration for a custom printer. Information on motors, lead screws, build plate size, and more must be supplied to this configuration tool and the custom firmware will be created to meet the specifications of the custom printer. Please note that testing and adjustments will be necessary for successful operation.
  ii. When the computer is connected to the printer motherboard and the firmware files are uploaded, the configuration files will be sent to and stored on the Motherboard’s micro-SD card.
  iii. Please note that when updating the firmware configuration of a custom printer, it is best to save a backup configuration file before making significant changes in the event an error may occur.
(b) Connect the Motherboard to a Computer
  i. Begin by connecting an Ethernet cord to the Ethernet terminal on the motherboard to an Ethernet terminal on your computer.
  ii. In the firmware configuration, select an IP address, netmask, and gateway for your printer using the M552, M553, and M554 commands. M552 defines the IP address according to the format 192.168.1.###. These last three unassigned digits, “###” can be assigned to any value between 2 and 255, as long as it differs from the last value of the computer IP address. M553 defines the subnet mask and should be set to 255.255.255.0. M554 defines the gateway and should be set to 192.168.1.1. Once this step is completed, save the configuration and restart the mainboard.
  iii. In your computer settings, navigate to Internet and Network settings.
  iv. Open the advanced network settings and select “Change adapter options.”
  v. Select the Ethernet connection for the printer, which is likely labeled “Unidentified network,” and open its properties menu.
  vi. Open the properties menu on “Internet Protocol Version 4 (TCP/IPv4).”
  vii. Select “Use the following IP address:” and assign the IP address to 192.168.1.1, the subnet mask to 255.255.255.0, and the default gateway to 192.168.1.2.
  viii. Press “Ok” and configure the network.
  ix. The RepRap Interface for the printer can now be accessed by typing the IP address assigned in the firmware configuration in a web browser.
  x. A video walkthrough of the IP address setup is included in the repository and can be referenced when following these steps.

**Fig. 11:**
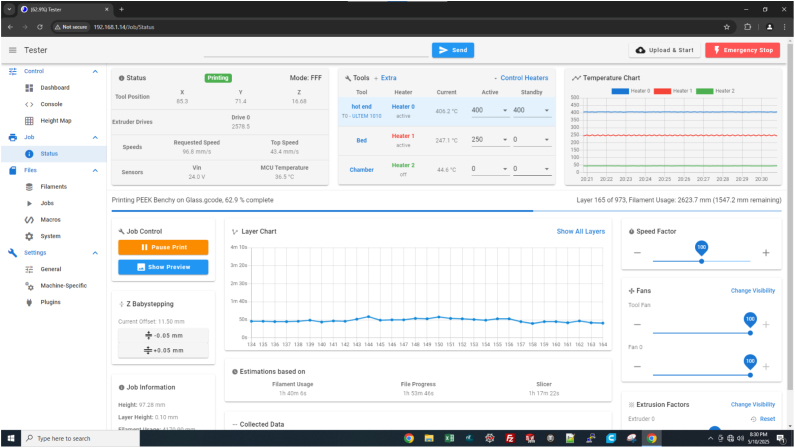
The custom RepRap firmware interface for printer operation and control during a PEEK print.

The RepRap firmware interface shown in Fig. 11 is used to operate and monitor the printer once it is turned on and activated with the power switch. Print progress, as well as layer height and tool temperatures can be monitored and adjusted during the printing process through this interface. Under the “Files” dropdown to the far left, the “System” tab can be selected to view the firmware configuration files and edit them on-demand or as needed to adjust the printer’s operation settings. Under this same dropdown, other files can be added such as filaments under the “Filaments” tab. Here a configuration file is made for loading and unloading each filament to dictate what loading and unloading temperatures the hotend should reach for a designated material. Also, the “Jobs” tab is where g-code files can be uploaded to the printer to queue print jobs and store commonly used job files. Under the “Control” dropdown tree, “Console” can be accessed to show any errors that may have been triggered to indicate a problem or fault during printing or system startup.

The firmware configuration used for this printer is available in the repository of this document. For more information on firmware configuration file editing, error codes, and more, the How-to guides on the Duet Documentation page are a great resource to understand the printer configuration code and fix most all firmware or wiring issues [33]. The Duet Forum is also a valuable resource for this work.

## 6 Operation instructions

(a) Plug an ethernet cord into the ethernet port on the Duet 2 Motherboard and connect the opposite end to a designated computer. (assuming steps for connecting motherboard to the computer have been completed to establish IP connection and all)
(b) Turn on the computer and open an empty tab to prepare for connection to the printer.
(c) Plug the 24 V DC power supply for the motherboard into a 120 V surge protector outlet.
(d) Turn on the motherboard power switch.
(e) Check for LEDs on the motherboard to light around the motors and the ethernet port, indicating that the board and its connected electronics are passing data and receiving power.
(f) In the address bar of the empty tab, type in the IP address assigned to the motherboard and access the firmware interface.
(g) Ensure all heated elements or tools are reading at room temperature.
(h) Ensure that the bed is level. Manually adjust if necessary.
(i) Plug the power cords for the chamber and bed heaters into a surge protector. Also, plug in the chamber fan to circulate heat.
(j) Make sure no major fluctuation in temperature (+/−5 degrees) occurs once they are plugged in. The temperature should be expected to fluctuate by +/−1 degree.
(k) Home all the axes with the CoreXY and Z-axis motors using the “Home All” command found on the firmware interface dashboard.
(l) Create and load a g-code file using PrusaSlicer or OrcaSlicer in “Jobs”, right click on the desired print job and hit “start print”.
Note Please note that when turning on the chamber heater, it will self-regulate its surface temperature to reach around 165°C, and the chamber temperature is dependent on the heater fan speed. Tuning this to reach a 100°C chamber temperature is required before starting to print. After calibration, it will take about 15 min to reach this temperature and be ready for printing. As the heat it spreads through the chamber, it will begin to heat the bed and nozzle, and as such, a fault may be thrown prematurely in either the nozzle or the bed. This is expected, and as such, there is no issue with the printer. To prevent this, begin heating all tools at the same time.

### 6.1 Recommendations for printing with ULTEM 1010 filament

(a) Ensure that the build plate has a minimum of four coats of Nano Polymer adhesive to maximize adhesion to the bed.
(b) For the experiments outlined in this paper, the ULTEM 1010 filament was kept at 10%–30% humidity in a filament dryer. This is the recommended storage for best results when printing with this material (add reference).
(c) A brim is needed for most parts as warping can happen even with properly applied Nano Polymer adhesive.
(d) The chamber temperature should be set to be around 100°C.
(e) The provided test files and print configurations for Ultem 1010, Ultem 9085, and PEEK act as a starting point, but individual optimization of extrusion width, layer height, and print speed are needed to maximize the print quality for your individual printer.

## 7 Validation & characterization

**Fig. 12:**
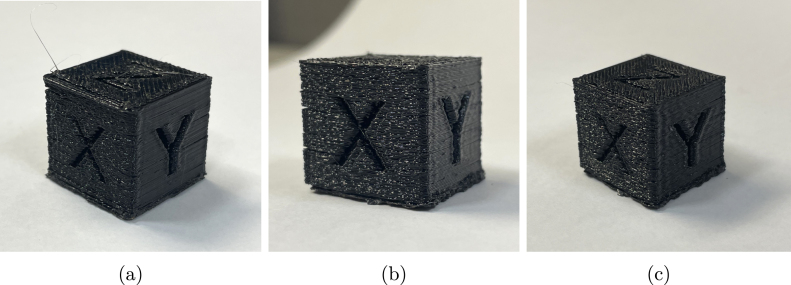
Isometric views of ULTEM 1010 XYZ calibration cubes.

With the configuration described in this paper, three XYZ calibration cubes of 20 × 20 × 20 mm were achieved with a ULTEM 1010 at a hotend temperature of 380°C, bed temperature of 200°C, ambient chamber temperature of 80°C with ULTEM 1010 material as shown in Figs. 12a, 12b, and 12c. These calibration cubes were printed and measured to estimate dimensional accuracy, precision, and repeatability. The results of these measurements are summarized in Table 4.

**Fig. 13:**
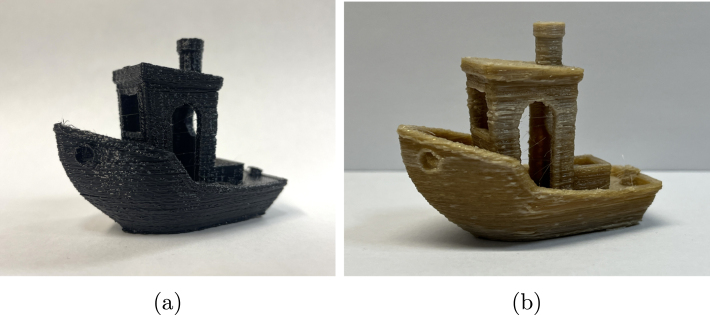
Isometric views of (a) ULTEM 1010 and (b) PEEK benchys.

Two benchys (Figs. 13a and 13b) were also created with this printer out of ULTEM 1010 and PEEK, respectively. After trial and error testing, it was found that ULTEM 9085 and PEEK (Fig. 13(b) were most easily and reliably fabricated with this printer in its current configuration as compared to ULTEM 1010 calibration specimens.

**Table 4:** Statistics for accuracy, precision, and repeatability of ULTEM 1010 calibration XYZ cubes.

Axes	Nominal dimension (mm)	Mean measured dimension (mm)	Bias (mm)	Percent error (%)	Standard deviation (mm)	Repeatability (%)
X	20	20.773	＋0.773	＋3.87	0.049	0.25
Y	20	20.607	＋0.607	＋3.04	0.187	0.94
Z	20	19.760	−0.240	−1.20	0.212	1.06

The heating elements were timed to evaluate their startup time for ULTEM printing temperatures from room temperature (20°C). The hotend requires around 5 min and 12 s, bed heater requires about 7 min and 12 s, and chamber heater requires 15 min to reach required temperatures for fabrication of high-performance polymers. The firmware and slicing configurations listed in the repository were used to generate this part and other geometries successfully.

During the final stages of development, the printer was optimized to improve print quality and determine optimal printing temperatures [34] All calibration parts discussed above were fabricated with the optimized printing parameters tailored to each material and geometry; the configuration files for these calibration prints and optimized parameters are available in the repository. In addition to the calibration prints discussed above, this system was used by Salisbury et al. to fabricate ASTM D638 Type IV tensile specimens from ULTEM 1010 to evaluate tensile strength [35]. These specimens had a mean tensile strength of 38.924 MPa with a sample standard deviation of 9.026 MPa. All tested specimens performed less than the requirements outlined in the material’s TDS value, with only three specimens performing within 5% of the target.

The printer was also used by Garrison et al. to fabricate ASTM D638 Type IV cupons, ASTM D7791-based cylinders, and a wing spar from ULTEM 9085 to assess its tensile and compressive fatigue behavior and compare its structural integrity with that of 6061 aluminum [36]. The ASTM D638 Type IV specimens were fabricated with raster orientations of 0° and 45° and tested in tension using an Instron 5882. The 0° specimens had a Young’s modulus of E=961±150MPa, an ultimate tensile strength of $\sigma_{\text{UTS}}=46.1\pm 6.4\;\mathrm{MPa}$, and a failure strain of ${ɛ}_{f}=0.057\pm 0.016$ mm/mm. In comparison, the 45° specimens showed reduced stiffness and strength, with E=396±94MPa, $\sigma_{\text{UTS}}=35.3\pm 8.9\;\mathrm{MPa}$, and an increased failure strain of ${ɛ}_{f}=0.103\pm 0.033$ mm/mm. Compressive fatigue tests were conducted on cylindrical samples based on ASTM D7791 at a frequency of 3 Hz and maximum stress levels of $\sigma_{\max}=30$–40MPa. Fatigue lives ranged from around $5\times 10^{3}$ to $1.3\times 10^{5}$ cycles. The wing spar was tested in a low-speed wind tunnel with a Reynolds number of Re≈8.0×104 and directly compared to an aluminum 6061-T6 spar. Measurements included six-axis force balance data and distributed fiber-optic strain sensing. These results established process-dependent tensile and compressive fatigue behavior for the printed material system and provided an experimentally grounded assessment of stiffness-to-mass tradeoffs governing aeroelastic strain response when substituting ULTEM 9085 for a conventional metal.

## 8 Conclusion & future work

An open-source high-temperature FFF 3D printer was successfully developed and tested with ULTEM 1010, ULTEM 9085, and PEEK high-performance polymers. The printer is capable of reaching the elevated temperatures needed to fabricate high-performance polymers with heating elements such as a hotend, heated bed, and chamber heater capable of 500°C, 200°C, and 120°C, respectively. ULTEM 1010 calibration cubes were used to determine the accuracy, precision, and repeatability of the printer under optimized printing parameters. Despite these optimizations, the results indicate that further calibration is required to improve dimensional accuracy and consistency before the system can reliably produce parts suitable for real-world applications. Overall, the printer developed in this study provides a viable framework for fabricating high-performance polymers, though additional process optimization and calibration are needed to achieve end-use quality and performance.

In future work, further optimization of printing parameters and calibration of the motion system (including motor tuning) should be performed. The integration of an automated bed-leveling system with optical methods is also recommended. In addition, replacing the existing bed heater with an off-the-shelf, PID-controlled solution would improve thermal stability and control. Finally, upgrading motion system components to metal CNC-fabricated parts would enhance performance at elevated chamber temperatures and reduce the risk of thermal degradation of polymer components in close proximity to the heated area.

## CRediT authorship contribution statement

**Charlotte Thompson:** Writing – review & editing, Writing – original draft, Validation, Methodology, Investigation, Data curation. **Luke Salisbury:** Writing – review & editing, Writing – original draft, Validation, Methodology, Investigation, Data curation. **Evan Garrison:** Writing – review & editing, Writing – original draft, Validation, Methodology, Investigation, Data curation. **Lillian DeJean:** Methodology, Investigation. **Santanu Kundu:** Writing – review & editing, Supervision, Resources, Conceptualization. **Matthew W. Priddy:** Writing – review & editing, Writing – original draft, Supervision, Resources, Project administration, Funding acquisition, Conceptualization.

## Ethics statement

No human or animal studies were conducted for this work.

## Declaration of competing interest

The authors declare that they have no known competing financial interests or personal relationships that could have appeared to influence the work reported in this paper.

The author is an Editorial Board Member/Editor-in-Chief/Associate Editor/Guest Editor for this journal and was not involved in the editorial review or the decision to publish this article.

## Appendix 9

### 9.1 Warnings

1. AC power terminals should not be left exposed especially when connected to power. Be sure these terminals are safely covered ad grounded at all times.
2. When changing filament, continue to extrude until nothing comes out. If filaments are mixed the hotend may be clogged beyond repair or discharge burnt material.
3. Do not leave the chamber heater plugged in when the printer is not in use as this is a ceramic heater and has the possibility of starting an electrical fire if left unattended.
4. Remove any part printed on the borosilicate glass bed as soon as it completed (especially for high temperature materials such as ULTEM and PEEK) because if left on the bed it may shatter.
5. Do not touch any of the heating elements or metal components of the printer while it is running at high temperatures without proper PPE such as welding gloves.
6. Do not remove the limiter from the chamber heater.
7. Use ABS or a more temperature resistant material for all 3D printed components on this printer, anything with less strength will melt and the printer will break or fail.

### 9.2 OSF repository contents

1. .csv file with all purchased parts/components
2. all CAD Files (STL, STEP, SLDPRT)
3. .cam files
4. firmware files
5. example test job files for validation

### 9.3 Consumables

1. linear bearings
2. nano polymer adhesive
3. gear grease
4. PC fan
5. hotend fan
6. belts
7. limit switches
8. glass plates

### 9.4 Needed tools for machining & construction

1. CNC
2. water jet
3. drill press
4. bandsaw
5. mill
6. 13 mm wrench or socket
7. 5 mm Allen key
8. 3 mm Allen key
9. 2 mm Allen key
10. 1.5 mm Allen key
11. 5/64 in Allen key
12. adjustable wrench
13. Phillips head screwdriver
14. ferrule terminal crimpers
15. fork terminal crimpers
